# Telemonitoring can assist in managing cardiovascular disease in primary care: a systematic review of systematic reviews

**DOI:** 10.1186/1471-2296-15-43

**Published:** 2014-03-07

**Authors:** Renee Purcell, Susan McInnes, Elizabeth J Halcomb

**Affiliations:** 1 School of Nursing and Midwifery, University of Western Sydney, Penrith, NSW, Australia; 2 School of Nursing & Midwifery, University of Wollongong, Wollongong, NSW, Australia

**Keywords:** Systematic review, Telemonitoring, Heart failure, Hypertension, Primary care

## Abstract

**Background:**

There has been growing interest regarding the impact of telemonitoring and its ability to reduce the increasing burden of chronic diseases, including chronic cardiovascular disease (CVD), on healthcare systems. A number of randomised trials have been undertaken internationally and synthesised into various systematic reviews to establish an evidence base for this model of care. This study sought to synthesise and critically evaluate this large body of evidence to inform clinicians, researchers and policy makers.

**Methods:**

A systematic review of systematic reviews investigating the impact of telemonitoring interventions in the primary care management of CVD was conducted. Reviews were included if they explored primary care based telemonitoring in either CVD, heart failure or hypertension, were reported in the English language and were published between 2000 and 2013. Data was extracted by one reviewer and checked by a second reviewer using a standardised form. Two assessors then rated the quality of each review using the Overview Quality Assessment Questionnaire (OQAQ).

**Results:**

Of the 13 included reviews, four focused on telemonitoring interventions in hypertension or CVD management and the remaining 9 reviews investigated telemonitoring in HF management. Seven reviews scored a five or above on the OQAQ evidencing good quality reviews. Findings suggest that telemonitoring can contribute to significant reductions in blood pressure, decreased all-cause and HF related hospitalisations, reduced all-cause mortality and improved quality of life. Telemonitoring was also demonstrated to reduce health care costs and appears acceptable to patients.

**Conclusion:**

Telemonitoring has the potential to enhance primary care management of CVD by improving patient outcomes and reducing health costs. However, further research needs to explore the specific elements of telemonitoring interventions to determine the relative value of the various elements. Additionally, the ways in which telemonitoring care improves health outcomes needs to be further explored to understand the nature of these interventions.

## Background

The growing burden of the group of diseases that comprise cardiovascular disease (CVD) on national healthcare systems is well recognised amongst healthcare professionals internationally. Despite a trend towards decreasing mortality, CVD remains the major cause of death worldwide [1]. In Australia, CVD accounts for a greater proportion of deaths than any other disease group, and accounts for the highest proportion of direct health care expenditure [2]. Improved acute care, advanced intervention techniques, early diagnostic procedures, and worldwide demographic ageing have resulted in an increasing number of patients living with chronic CVD [3,4]. As a result, chronic heart failure (HF) is becoming an increasing health issue [5]. Almost half the individuals admitted to hospital for HF are re-admitted within 6 months [6,7]. These frequent hospital re-admissions contribute significantly to the high costs of HF management [6,7]. Up to 70% of HF cases, are preceded by hypertension [5]. Implementation of evidence based hypertension management has the potential to reduce the prevalence of HF and, subsequently, reduce HF burden. Given the interrelationship and progressive nature of cardiac disease in primary care, there are advantages in considering the impact of management strategies across the disease silos.

The increasing burden associated with CVD necessitates the investigation of innovative models to provide evidence-based care to promote early identification of exacerbations and early intervention to minimise their severity. Among the available models is telemonitoring, which consists of remote monitoring of patients to enable clinicians to intervene when there is evidence of clinical deterioration in an attempt to avoid hospitalisation [8]. Telemonitoring moves patient care out of a clinical setting into the patient’s home by utilising telecommunications technologies such as the Internet, telephone, or videoconferencing to transmit physiological data and information about current symptoms from the patient to health care professionals [8]. Shifting the burden of care to the patient’s home also facilitates supported self-care, giving the patient enhanced autonomy and control of their health care [8]. Telemonitoring programs can be flexible, individually tailored, and have the potential to provide access to specialist care for a larger number of patients across a much greater geography when compared to usual care [8-10].

Currently the literature reports multiple systematic reviews investigating the effectiveness of telemonitoring in both HF and its antecedent, hypertension. Given the large amount of literature available, a systematic review of reviews was conducted to synthesise and critically evaluate this current Level 1 evidence around the use of telemonitoring in the primary care setting.

## Methods

This review was conducted using the Preferred Reporting Items of Systematic Reviews Meta-Analyses (PRISMA) guidelines [11]. A systematic review design was selected to limit any bias in the selection and reporting of evidence.

### Search strategy

A comprehensive and systematic search was undertaken using CINAHL, MEDLINE and the Cochrane Library electronic databases. The search strategy included the Medical Subject Headings (MeSH) terms; telehealth, telemedicine, heart failure, hypertension, cardiovascular disease and systematic review. As a relatively new form of technology, reviews were retrieved if they were published between 2000 and 2013. Secondary searching of the reference lists of retrieved papers and of the Internet via the Google scholar search engine was undertaken to identify any additional reviews that met the inclusion criteria.

### Selection procedures

Papers were retrieved based on whether the title and abstract or, if required, the full manuscript met the inclusion criteria for this review. Papers identified through the search were assessed based on the inclusion and exclusion criteria by two independent reviewers (RP and EH). Where there was discrepancy, the reviewers discussed the issues and reached consensus.

### Inclusion criteria

Systematic reviews were included if they met the following inclusion criteria. Firstly, the paper needed to report a systematic review or meta-analysis of original intervention studies. Reviews that did not meet the definition of a systematic review or meta-analysis, or were expert commentaries were not included [8]. Secondly, included reviews needed to explore the impact of telemonitoring on the health outcomes of adults with known CVD as distinct from those with other chronic diseases. An a priori decision was made to not include studies with paediatric samples. Thirdly, given the focus on telemonitoring as distinct from home monitoring, reviews were excluded if there was no evidence that home based measures were transmitted to a healthcare provider. Finally, due to resource constraints and ease of access, reviews that were published in any language other than English, or reviews that were unpublished were excluded from the analysis.

### Data extraction

Two authors (RP and EH) developed a standardised form for extracting data and relevant information from the 13 included reviews. Whilst developed specifically for this review, the form was based on tools used in other systematic reviews and systematic reviews of reviews [12,13]. The form contained 10 categories regarding the characteristics and results of the included reviews. All data were initially extracted by one author (RP), this extraction was then checked by a second author (EH) for accuracy. Where differences were identified, the authors discussed issues and reached a consensus decision.

### Methodological quality

A two-stage process was undertaken to evaluate both the type of evidence contained in each included review, and the quality of the review process used. In the first stage of this process, the level of evidence was graded using the National Health & Medical Research Council (NHMRC) hierarchy (Table 1). Whilst systematic reviews of randomised controlled trials are considered the highest level of evidence on this hierarchy, other reviews are ranked only as high as their included studies [14].

**Table 1:** **NHMRC hierarchy of evidence**[14]

**Level of evidence**	**Descriptor**
I	Systematic review of randomised controlled trials
II	Randomised controlled trial
III	Pseudo-randomised controlled trial, comparative study with or without concurrent controls
IV	Case series with either post-test or pre-test/post-test outcomes

In the second stage of this process, the quality of included systematic reviews and meta-analyses were assessed using the component and total scores from the Overview Quality Assessment Questionnaire (OQAQ). The OQAQ has been previously validated as a measure of quality in research reviews [15,16]. Two reviewers independently scored each paper using OQAQ (RP and SM) and any disparities were discussed with a third reviewer (EH) until consensus was reached.

## Results

**Figure 1:**
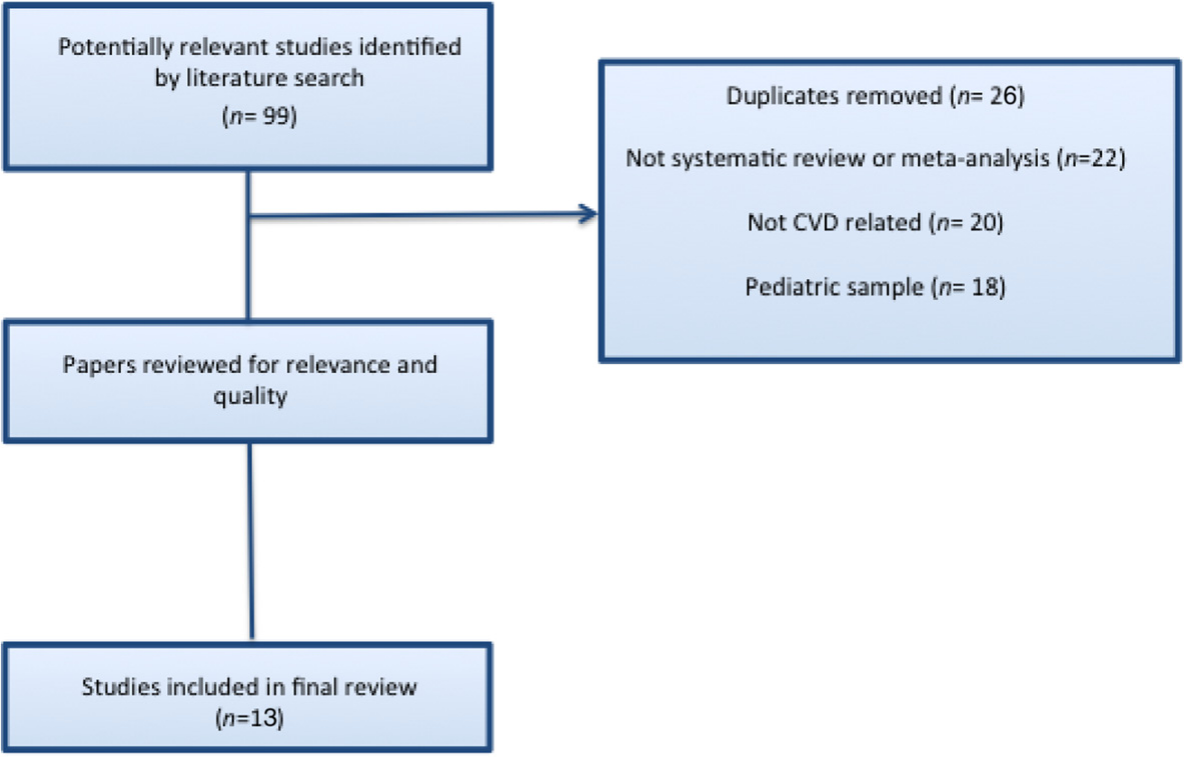
Process of review selection.

Ninety-nine papers were identified by the search strategy and assessed against the review inclusion criteria. All reviewers agreed that 13 papers met the inclusion criteria for the review. Papers were excluded if they did not distinguish individuals with CVD from those in other disease groups, the intervention did not involve information transmission between the consumer and health provider or did not report a formal systematic review (references to these papers can be provided on request). The flowchart in Figure 1 outlines the process for selecting included papers.

Of the 13 included reviews, nine were graded as Level 1 on the NHMRC Hierarchy [8,10,17-23]. The remaining reviews included a combination of RCTs, observational, descriptive, case series and/or non-randomised controlled studies, therefore, were considered to be Level II to IV evidence [24-27]. Seven reviews [8,10,17,18,23,26,28] scored five or above on OQAQ, indicating minor or no methodological flaws [15] (Table 2). Reviews that failed to score above a five predominantly did so due to the poor assessment of validity in primary studies and/or the authors failing to use a validated quality scoring system. The quality features of included systematic reviews are presented in Table 2.

**Table 2:** OQAQ Scores for the methodological quality of included reviews

**Quality criteria**	**Clark et al. **[10]	**Neubeck et al. **[18]	**Inglis et al. **[8]	**Martínez et al. **[26]	**Agarwal et al. **[17]	**Klersy et al. **[23]	**Chaudhry et al. **[21]	**Giamouzis et al. **[22]	**Seto **[27]	**Verberk et al. **[20]	**Louis et al. **[24]	**Maric et al. **[25]	**Omboni et al. **[19]
**Search methods used to find evidence stated**	3	1	3	3	3	3	3	2	3	3	3	2	3
**Search for evidence reasonably comprehensive**	3	3	3	2	2	2	2	2	2	2	2	2	2
**Criteria used for deciding which studies to include reported**	3	3	3	2	3	2	2	3	3	3	2	3	3
**Bias in the selection of studies avoided**	3	3	3	2	3	3	2	3	3	3	2	3	3
**Criteria used for assessing validity of included studies reported**	3	3	3	3	2	2	2	1	1	1	1	1	1
**Validity of included studies assessed appropriately**	3	3	2	3	2	2	2	1	1	1	1	1	1
**Methods used to combine the findings of studies reported**	3	3	3	3	3	3	3	2	3	3	2	2	3
**Findings of studies combined appropriately**	3	3	3	3	3	3	3	2	3	3	2	2	2
**Conclusions made by authors supported by analysis**	3	3	3	3	3	3	2	3	3	2	3	2	3
**Overall Quality Score**	**7**	**7**	**6**	**6**	**5**	**5**	**4**	**3**	**3**	**3**	**3**	**2**	**2**

As can be seen in Tables 3 and 4, 9 reviews investigated telemonitoring for heart failure [8,10,21-27], three focused on telemonitoring interventions for hypertension [17,19,20] and a single review looked at risk factor reduction in CVD [18]. The number of studies synthesised in the included reviews varied between 9 [20,21] and 56 [25] studies. Whilst the number of included participants was not accurately reported in two reviews [24,26], in the remaining reviews the number of participants ranged from 2,401 to 9,946 [8,10,17-22,25,27,28].

**Table 3:** Summary table of included reviews - hypertension

**Citation**	**NHMRC level of evidence**	**Included studies**	**Total participants**	**Participants condition**	**Inclusion criteria**	**Intervention**	**Outcomes**	**Results**	**Conclusion**
Agarwal et al. [17]	I	37	9446	Hypertension	• Published between 1966-2010	Home BP monitoring compared to a control group.	• BP - diastolic, systolic and mean arterial • Medication use	Compared with clinic based measurements, home based BP monitoring; • Improved systolic BP (SMD −2.63 mmHg, 95% CI −4.24 to −1.02; 22 studies) • Improved diastolic BP (SMD - 1.68 mmHg, 95% CI −2.58 to −0.79 mmHg; 22 studies) • Improved mean arterial pressure (SMD −4.0 mmHg, 95% CI −6.22 to −1.79 mmHg; 3 studies) • Reduced medication use (RR 2.02, 95% CI 1.32 to 3.11; 10 studies) • Reduced therapeutic inertia (RR 0.82, 95% CI 0.68 to 0.99; 15 studies) • Led to no greater increase in medication (RR 0.94, 95% CI 0.75 to 1.19; 12 studies)	• Compared with clinic BP monitoring alone, home BP monitoring had the potential to overcome therapeutic inertia [no change in medication]. • Lead to a small but significant reduction in systolic and diastolic BP. • Hypertension management with home BP monitoring can be enhanced when used with telemonitoring.
Neubeck et al. [18]	I	11	3145	Coronary Heart Disease	• English language • Published between 1990-2008	Intervention involved home monitoring with 50% patient provider contact for risk factor modification and advice/counselling for CHD patients	• All-cause mortality • Modifiable risk factors including cholesterol (and associated measures), BP, BMI, Smoking Status, Physical Activity • Quality of life • Cost	Compared to the control group the evidence suggests that the intervention group had; • Reduced total cholesterol (WMD 0.37 mmol/L, 95% CI: 0.19 to 0.56, 9 studies) • Reduced low-density lipoprotein cholesterol (WMD 0.41 mmol/L, 95% CI: 0.36 to 0.56, 4 studies) • Reduced systolic blood pressure (WMD 4.69 mmHg, 95% CI 2.91 to 6.47, 7 studies) • Reduced risk of smoking (RR 0.83, 95% CI: 0.70 to 0.99, 7 studies)	• Telemonitoring interventions provided effective risk factor reduction and secondary prevention in patients with CHD. • Telemonitoring could increase the uptake of formal secondary prevention by those who do not access cardiac rehabilitation, and narrow the current gap between evidence and practice.
						• Telephone based telemonitoring – 9 studies			
						• Internet based telemonitoring – 2 studies			
Omboni et al. [19]	I	12	5044	Hypertension	• English language • Published between inception - 2010	Home BP monitoring with data being automatically transferred compared to control group.	• Change in BP (diastolic, systolic and normalisation) • Medication	Compared to controls the overall effect of home BP monitoring was; • Improved office systolic BP (5.64 mm Hg, 95% CI: 7.92 to 3.36 mm Hg, 11 studies) • Improved office diastolic BP (2.78 mm Hg, 95% CI: 3.93 to 1.62 mm Hg, 11studies) • Improved ambulatory systolic BP (2.28 mm Hg, 95% CI: 4.32 to 0.24 mm Hg; 3 studies) • Improvement in BP control (RR 1.31, 95% CI: 1.06 1.62, 5 studies)	• Home blood pressure telemonitoring may represent a useful tool to improve blood pressure control but well-designed large-scale trials are still needed to demonstrate its clinical usefulness.
Verberk et al. [20]	I	9	2662	Hypertension	• English language • No restriction on dates reported	Home BP transmitted via telephone, internet, modem or mail.	• BP - diastolic, systolic • Medication	• Increased use of antihypertensive medications (WMD +0.22, 95% CI: +0.02, +0.43, 5 studies) Compared with usual care, home based BP monitoring; • Reduced systolic BP (5.19 mmHg, 95% CI 2.31 to 8.07; 9 studies) • Reduced diastolic BP (2.11 mmHg, 95% CI 0.52 to 3.69; 9 studies) • There was no significant difference between groups in the number of patients that reached their target blood pressure (3 studies)	• Telecare led to a greater decrease in systolic and diastolic blood pressure than usual care. For systolic blood pressure, this decrease was greater in trials without treatment modification.

**Table 4:** Summary table of included reviews – heart failure

**Citation**	**NHMRC level of evidence**	**Included studies**	**Total participants**	**Inclusion criteria**	**Intervention**	**Outcomes**	**Results**
Chaudhry et al. [21]	I	9	3582	• English language • Published between 1966-2006	Nurse-led telephone symptom monitoring (no meta analysis) – 5 studies Automated monitoring of signs & symptoms – 1 study Automated physiological monitoring – 1 study Comparisons of two or more methods of telemonitoring (no meta-analysis) – 2 studies	• All cause and HF mortality • All cause and HF admissions • Cost	• Reduced all-cause hospitalisation (47%) (1 study) • Reduced HF hospitalisations (2 studies) (20-50% reduction) • No significant difference in HF hospitalisations (2 studies) • Reduced emergency room visits (95% CI 0.36-0.80)(1 study) • Reduced mortality (1 study) • Reduced health care costs ($1000 less per patient) (1 study) • No significant difference in all-cause hospitalisations (1 study) • Reduced mortality (56% - 95% CI 0.22-0.85) (1 study) • Reduced HF hospitalisations (1 study) (40% - 95% CI 0.45-0.82) • Reduced health care costs ($276705 less 6-month cumulative readmission charges in the intervention group) (1 study) • Both physiologic monitoring and regular nurse telephone calls showed improved mortality and hospitalisation rates compared to usual care (1 study). • No between group differences in mortality and hospitalisation rates between physiologic monitoring and regular nurse telephone calls (12.7% vs 15.9%) (1 study). • Both video conferencing and nursing support by telephone showed reduced 6-month HF readmission charges compared to usual care (1 study). • No between group differences in 6-month HF readmission charges were seen between video conferencing and nursing support by telephone (1 study).
Clark et al.[10]	I	14	4264	• English language • Published between 2002-2006	Telemonitoring – 4 studies Structured telephone support – 9 studies Telemonitoring and structured telephone support – 1 study	• All-cause admissions • HF admissions • Quality of life • Acceptability • Cost • All-cause mortality	• Both interventions were associated with a statistically significant 20% reduction in all-cause mortality (RR 0.80, 95% CI: 0.69 to 0.92; 14 studies)
							• A decrease in all-cause mortality was more pronounced with telemonitoring (RR 0.62, 95% CI: 0.45 to 0.85; 4 studies) than with structured telephone support (RR 0.85, 95% CI: 0.72 to 1.01; 9 studies) • HF related hospitalisation was significantly reduced by 20% through remote monitoring programmes (RR 0.79, 95% CI 11%-31%). • None of the 8 studies that reported all cause admission to hospital reported a statistically significant result. The pooled estimates also did not show significant benefit. • 3/6 trials that investigated quality of life reported a significant and substantial improvement. • 3/4 trials of structured telephone support reported lower healthcare costs. • 4 trials reported acceptability of the intervention to patients.
Giamouzis et al. [22]	I	12	3,877	• English language • Published between 1991 and November 2011 • Follow-up of at least 6 months • At least 1 device to measure and transmit physiological data	Intervention involved recording physiological data by portable devices, and transmitting data remotely to a server.	• CVD related mortality • All-cause mortality • Hospitalisation/Readmissions • Cost	Compared to controls the telemonitoring groups had: • Reduced hospitalisation rates that reached statistical significance (3 studies) • Reduced hospitalisation rates without reaching statistical significance (4 studies). • Statistically significant reduced all-cause mortality (3 studies). • Fewer reported deaths, however these results were not statistically significant (5 studies) • Evidence for costs associated with telemonitoring were mixed with two studies finding cost reductions and one study finding increased costs. • In four studies there were more re-hospitalisations in telemonitoring groups compared to usual care groups, but these findings were either not statistically significant or significance was not reported.
Inglis et al.[8]	I	25	8323	• Published between 1999 – 2008	Telemonitoring (transfer of daily data) – 11 studies Structured Telephone support – 16 studies Both interventions – 2 studies	• HF and all-cause admissions • Quality of life • Acceptability • Cost • All-cause mortality • Length of stay	• Telemonitoring reduced all-cause mortality (RR 0.66, 95% CI: 0.54–0.81; 11 studies) • Structured telephone support showed a non-significant trend towards reduced all-cause mortality (RR 0.88 95% CI: 0.76– 1.01; 15 studies) • Both telemonitoring (RR 0.79, 95% CI: 0.67–0.94; 4 studies), and structured telephone support (RR 0.77, 95% CI 0.68–0.87; 13 studies) reduced chronic heart failure related hospitalisations • Both interventions improved quality of life, reduced costs, and were acceptable to patients • 1/6 studies reported a statistically significant reduction in length of stay, with a further 2 studies reporting a non-significant reduction
Klersy et al. [23]	I	21	5715	• Published before September 2009 • RCTS reporting hospitalisation and LOS data	Either structured telephone monitoring or technology assisted monitoring – collectively referred to as remote patient monitoring.	• Hospitalisations • LOS • Cost • Quality of life	• Remote patient monitoring was associated with significantly fewer hospitalizations for HF (incidence rate ratio: 0.77, 95% CI 0.65–0.91, P < 0.001) (18 studies) • Remote patient monitoring was associated with significantly fewer hospitalizations for any cause (incidence rate ratio: 0.87, 95% CI: 0.79–0.96, P = 0.003) (18 studies) • LOS was not different between remote patient monitoring and usual care for either HF hospitalisations (95% CI 20.12–0.13, P = 0.88) or all-cause hospitalisation (95% CI 20.18–0.02, P = 0.83) (12 studies). • RPM reduced costs between 300 to 1000 euros • RPM was associated with a gain of 0.06 quality-adjusted life years – 0.02 due to reduced mortality and 0.04 due to reduced hospitalisations
Louis et al. [24]	III^1^	24	Not reported accurately	• English language • Published between 1966-2002	Home monitoring using specialised devices in conjunction with a telecommunication systems.	• All-cause mortality • HF admissions • Length of stay • Quality of life • Acceptability • Compliance • Cost • ED presentations	Observational studies suggested that telemonitoring: • Reduced hospitalisation (10 studies) and readmission rates (2 studies) • Reduced length of stay (4 studies) • Reduced ED presentations (2 studies) • Reduced inpatient costs (1 study) • Was acceptable to patients (3 studies), patients were highly satisfied (>86%)(2 studies) and improved quality of life (1 study). Compared with usual care telemonitoring RCTs: • Reduced hospitalisation (2 studies) and readmission rates (1 study) • Reduced mortality (1 study)
							• Reduced length of stay (1 study)
							• Improved quality of life and high patient satisfaction (1 study)
Maric et al. [25]	IV^2^	56	--	• English language • Published before August 2007	Device-based technologies - 16 studies Telephone touch-pads - 12 studies Video-consultation-based studies - 3 studies Website-based telemonitoring - 5 studies Combined modalities - 21	• Hospitalisation • Quality of life • Medication • Cost • Length of stay	• Decreased hospitalizations (8 studies) • Improved QOL (5 studies) • Fewer re-hospitalizations and combined events (1 study) • Reduced time to target drug dosage (1 study) • No significant changes (1 study) • Change in mood (1 study) • Improved QOL (1 study) • Reduced hospital length of stay (1 study) • Increased hospital length of stay (1 study) • Decreased hospitalizations (7 studies) • Reduced costs (6 studies)
Martínez et al. [26]	IV^3^	42	Not reported accurately				
				• English and Spanish language • Published between 1951-2004	Home monitoring of HF patients using peripheral devices for measuring and automatically transmitting data.	• Cost • Acceptability • Health status • Hospital admissions • Length of stay • Quality of life • Feasibility/viability	Compared to the control groups the evidence suggests that telemonitoring; • Improved quality of life (12 studies) • Reduced length of hospitalisation (12 studies) • Reduced mortality (4 studies) • Reduced costs (9 studies) • Reduced unattended emergencies (1 study) • Equipment easy to use (5 studies)
Seto [27]	III^4^	10	586	• English language • Published between inception – April 2010	Telemonitoring systems with a component of home physiological measurements.	• Cost	• 9/10 studies analysed direct healthcare system costs. 1/10 study investigated direct patient costs. • All the studies found cost reductions from telemonitoring compared to usual care, ranging between 1.6% and 68.3% • Cost reductions were predominantly attributed to reduced hospitalisation expenditures. • A 3.5% lower direct patient costs was identified, related to patient travelling. • 55% of patients were willing to pay $20 to use telemedicine and 19% were willing to pay $40.

^1^RCT– 6 studies, Non-randomised – 12 studies, Observational- 6 studies.

^2^RCT- 23 Studies, Non Randomised – 10 Studies, Pre-post – 15 Studies, Feasibility – 1 Study, Unknown design – 8.

^3^RCT – 13 studies, Non-randomised – 10 studies, Clinical series or descriptive studies – 19 studies.

^4^RCT- 5 studies, Non-randomised - 4 studies, Survey – 1 study.

Definitions of telemonitoring varied, with some reviews of hypertension management only citing studies that used telemonitoring to transmit physiological data [20,22] and other reviews included studies of telemonitoring combined with additional support, such as counselling, and/or education [8,10,17-19,21,24-28]. In some reviews it was possible to compare outcomes between various types of interventions [8,21,25], whilst in others various interventions were combined in the analysis [23]. This variation is a reflection of the current state of the literature and the significant variety of operational definitions of telemonitoring between studies included in the reviews.

A diverse range of outcomes was measured across the reviews. Those reviews which investigated hypertension primarily used blood pressure and medication use as outcomes (Table 3), whilst those investigating HF used a combination of outcomes including, mortality, hospital admissions, quality of life, cost, acceptability (Table 4). All reviews reported benefits associated with telemonitoring, however the significance of the identified benefits differed between studies and reviews. No review reported negative effects of telemonitoring or harm to patients. All reviews noted significant variance between included studies with four reviews citing statistically significant heterogeneity [8,18,19]. It is essential that this be considered in the interpretation of these data.

### Synthesis of reviews of hypertension & CVD management

Table 3 provides a summary of the four included reviews that focused on telemonitoring in hypertension and CVD management.

#### Blood pressure

The four reviews which investigated hypertension and CVD management all used blood pressure as an outcome measure [17-20]. The operational definition of blood pressure differed across reviews with all reviews reporting systolic blood pressure [17-20], three reporting diastolic pressure [17,19,20], one review reporting mean arterial pressure [17] and another reporting blood pressure normalisation [19]. All reviews reported significant reductions in blood pressure with the various interventions (Table 3). Given the concomitant changes in extraneous variables such as medication use and lifestyle risk factors that occurred around the interventions, it is difficult to ascertain which specific aspects of the intervention led to the improvements in blood pressure seen within these reviews and included studies.

#### Medication use

Three reviews which investigated hypertension and CVD management reported medication use as an outcome measure [17,19,20]. There was some variation in finding of medication use between reviews. Agarwal et al. [17] reported that 10 studies demonstrated reduced medication use (RR 2.02, 95% CI 1.32 to 3.11) and 12 studies reported no greater increase in medication (RR 0.94, 95% CI 0.75 to 1.19). In contrast, Omboni & Guarda [19] reported five studies that demonstrated increased use of antihypertensive medications (WMD +0.22, 95% CI: +0.02, +0.43). It should be noted that this increase in antihypertensive use may have been a positive finding if patients had been sub-optimally medicated and were now receiving best practice pharmacotherapy.

In their review, Verberk et al. [20] compared the outcomes of those who had their antihypertensive treatment modified and those who did not have modified treatment during the study in both the intervention and treatment groups. This comparison demonstrated that treatment modification was associated with significantly lower systolic BP compared to non-modification (5.1 mmHg ± 2.9 mmHg lower).

### Synthesis of reviews of heart failure management

Table 4 provides a summary of the nine included reviews that focused on telemonitoring in HF management.

#### Hospital admission

Eight of the reviews of HF management used hospitalisation as an outcome measure [8,10,21-26]. All these reviews reported that telemonitoring was associated with reduced hospitalisations [8,10,21-27]. The reduction in hospitalisation was reportedly as high as 50% in one review [21]. However, three reviews reported included studies that did not demonstrate a statistically significant reduction in hospitalisation rates [10,21,22]. Indeed in their review, Giamouzis et al. [22] identified four included studies in which the telemonitoring group had more rehospitalisation’s than the usual care group, although these findings were either non-significant or the significance was not reported.

In terms of HF related hospitalisations, Inglis et al. [8] reported that both telemonitoring and structured telephone support reduced the number of patients having a HF related admission to hospital (structured telephone support RR 0.77, 95% CI 0.68 to 0.87, P < 0.0001 and telemonitoring RR 0.79, 95% CI 0.67 to 0.94, P = 0.008). Similarly, Chaudhry et al. [21] found that both video conferencing and regular nurse phone calls reduced 6-month HF related admissions to hospital compared to usual care. However, there were no between group differences between video conferencing and regular nurse support.

#### Length of stay

Length of stay (LOS) was reported as an outcome in five included reviews [8,23-26]. However, in three of these reviews, fewer than six included studies reported LOS as an outcome [8,24,25]. In these three reviews all but one study reported reduced length of hospital stay following the intervention [25].

The other two reviews included 12 studies each that reported LOS as an outcome, although had conflicting findings. The recent review published by Klersy [23] concluded that length of stay was not different between remote patient monitoring (combined telemonitoring and structured telephone support) and usual care for either HF (95% CI 20.12–0.13, P = 0.88) or all-cause hospitalisation (95% CI 20.18–0.02, P = 0.83). However, the older review by Martínez et al. [26] reported that telemonitoring reduced length of hospitalisation.

#### Mortality

Six reviews of HF management used all-cause mortality as an outcome measure [8,10,21,22,24,26]. As can be seen from Table 5, a range of interventions improved mortality. In their comparative reviews, both Clark et al. [10] and Inglis et al. [8] demonstrated that all-cause mortality was more significantly reduced in studies using telemonitoring rather than structured telephone support.

**Table 5:** Summary of all-cause mortality

**Reference**	**Intervention**	**Results**
Chaudhry et al. [21]	Automated monitoring of signs & symptoms	56% reduced mortality (1 study)
		95% CI 0.22-0.85
Clark et al. [10]	Telemonitoring – 4 studies	20% reduction in all-cause mortality (RR 0.80, 95% CI: 0.69 to 0.92; 14 studies)
	Structured telephone support – 9 studies	Decrease in all-cause mortality more pronounced with telemonitoring (RR 0.62, 95% CI: 0.45 to 0.85; 4 studies) than with structured telephone support (RR 0.85, 95% CI: 0.72 to 1.01; 9 studies)
	Telemonitoring and structured telephone support – 1 study	
Giamouzis et al. [22]	Telemonitoring	Statistically significant reduced all-cause mortality (3 studies).
Inglis et al. [8]	Telemonitoring (transfer of daily data) – 11 studies	Telemonitoring reduced all-cause mortality (RR 0.66, 95% CI: 0.54–0.81; 11 studies)
	Structured Telephone support – 16 studies	Structured telephone support showed a non-significant trend towards reduced all-cause mortality (RR 0.88 95% CI: 0.76– 1.01; 15 studies)

#### Quality of life

Six reviews of telemonitoring in HF reported quality of life (QOL) as an outcome measure [8,10,23-26]. All of these reviews described some studies that had demonstrated improved quality of life (Table 1). There was limited information provided about the measures used to evaluate QOL. Only Klersy et al. [23] reported quality of life in terms of quality adjusted life years (QALYS). This review calculated that remote patient monitoring was associated with a gain of 0.06 QALYs – 0.02 due to reduced mortality and 0.04 due to reduced hospitalisations [23].

#### Cost

Two reviews of telemonitoring in HF focused on cost as an outcome measure [23,27]. Both of these reviews concluded that telemonitoring reduced costs compared to usual care. This reduction in costs was attributed to the reduction in hospital admissions. Seto [27] also reported a 3.5% saving in direct patient costs as a result of the reduced need for patients to travel to services. Additionally, Seto [27] identified that over half of patients were willing to make a financial contribution to allow them to use telemedicine.

## Discussion

This systematic review of systematic reviews found that the use of telemonitoring for patients with hypertension and HF was associated with multiple benefits. The reviews of telemonitoring for hypertension demonstrated that various telemonitoring interventions were able to effect significant reductions in blood pressure. The reviews of telemonitoring for HF demonstrated a reduced risk of mortality [8,10,21,22,24,26], fewer hospitalisations [8,10,21,22,24-27], reduced health care costs [23,27] and improved quality of life compared to usual care [8,10,24-26]. It has been reported in two syntheses of the effectiveness of telemonitoring across disease groups that the evidence supporting the efficacy of telemonitoring is most favourable for HF and hypertension compared to other forms of chronic disease [29-31].

A key limitation in the included reviews is the heterogeneity of the interventions reported in included studies and reviews [8,18,19]. Telemonitoring interventions are frequently multi-dimensional, containing a range of elements including the transmission of physiological data, coaching, telephone support, video-consultations, nurse interventions and web based communications [30]. The rapid technological advancements that have been seen in the last decade may also impact on the ability to compare older and newer studies using different technology [30]. Whilst some reviews reported comparisons between the various types of telemonitoring interventions, in others it was more difficult to differentiate the outcomes from the various interventions. Further research needs to tease out the specific aspects of telemonitoring interventions that are essential to improving health outcomes.

A further limitation was the lack of reporting of or use of unvalidated outcome measures. Whilst some reviews reported the range of outcomes measures used in included studies, others provided limited data on which to evaluate how the various outcomes were measured.

A final limitation is that, as a systematic review of reviews, this paper only included studies that had been reported within systematic reviews. This means that some more recent research is not included in the analysis. Whilst this recent literature includes several papers which affirm the value of telemonitoring [32-35], it also includes some large trials of telemonitoring in HF and hypertension which report negative results [36-39]. These divergent findings highlight the need for future research to more carefully interrogate how and why telemonitoring interventions work to improve outcomes.

Whilst the specific elements of the interventions that underpinned the positive outcomes are not clear, it is postulated that the benefits of telemonitoring reflect a combination of improved implementation of and adherence to guideline therapies, early identification of complications, and a positive impact on patient psychology [8,10]. Wotton [31] asserts that telemedicine is effective as it facilitates integration and case-management within chronic disease, both strategies well known to improve guideline adherence and early intervention. Further investigation into how telemedicine can best be integrated into existing clinical primary care to enhance case-management will assist in developing new models of care. Future research exploring the impact of telemonitoring on adherence to treatment guidelines and early identification of exacerbation, will increase our understanding of how telemonitoring interventions improve outcomes.

The benefits of telemonitoring can also be attributed to patient empowerment and its flow on effects [40]. Providing patients with the tools and education to monitor their own symptoms, vital signs, and fluctuating medication needs, empowers the patient to take an active role in their own healthcare. Improved self-management has long been recognised as a strategy to enhance outcomes in chronic disease. Given that patients largely find telemonitoring acceptable and easy to use [8,10,24,26,41-43], further research focusing on the impact of telemonitoring on self-management and the use of telemonitoring as a patient education tool would inform new models of care [44].

## Conclusions

In summary, this systematic review of systematic reviews found that telemonitoring has the potential to reduce the burden associated with hypertension and HF in primary care. However, further well-designed research is required to facilitate our understanding of how this intervention improves various outcomes and to allow the essential components of a telemonitoring intervention to be identified.

## Competing interests

The authors declare that they have no competing interests.

## Authors’ contributions

EH conceived the study, oversaw the conduct of the review and participated in the data analysis and drafting of the manuscript. RP and SM completed the data extraction and quality assessments of included papers as well as participating in the manuscript preparation. All authors read and approved the final manuscript.

## Pre-publication history

The pre-publication history for this paper can be accessed here:

http://www.biomedcentral.com/1471-2296/15/43/prepub
